# Square beams for optimal tiling in TEM

**DOI:** 10.21203/rs.3.rs-3220524/v1

**Published:** 2023-08-09

**Authors:** Eugene YD Chua, Lambertus M Alink, Mykhailo Kopylov, Alex de Marco

**Affiliations:** Simons Electron Microscopy Center, New York Structural Biology Center, New York, NY 10027

**Keywords:** TEM, cryo-EM, square beam, TEM tiling

## Abstract

Imaging large fields-of-view at a high magnification requires tiling. Transmission electron microscopes typically have round beam profiles; therefore, tiling across a large field-of-view is either imperfect or results in uneven exposures, which is a problem on dose-sensitive samples. Here we introduce a square electron beam that can be easily retrofitted in existing microscopes and demonstrate its application showing it can tile nearly perfectly and deliver cryo-EM imaging with resolution comparable to conventional setups.

In transmission electron microscopy (TEM) of dose-sensitive specimens such as vitrified biological material, pre-exposure of areas to the beam attenuates the attainable resolution (Baker & Rubinstein, 2010). Cryo-TEM imaging requires balancing capturing high-resolution information through high magnifications against having a sufficiently large field of view to image the entire region of interest. Since the illumination profile (which we call here “beam” or “beam profile” for simplicity) of TEMs is round, tiling across a large field of view encounters the circle packing problem, wherein circles cannot be perfectly tiled. Even with an ideal modern imaging setup with fringe-free imaging (FFI) (Konings et al., 2019; Weis & Hagen, 2020) and a square sensor, the sensor will only capture ~69% of the area illuminated by the tightest possible round beam (Figure 1 and Supplementary Figure 1). The electron beam will damage the remaining illuminated but unimaged area, which will no longer contain high-resolution information when next imaged. This is a well-known limitation in montage tomography of vitrified specimens, and while data collection schemes that account for overlapping exposures exist (Peck et al., 2022; Yang et al., 2022), the illumination across multiple exposed areas remains non-uniform.

One solution to the problem of imperfect tiling with round beams is to use a square electron beam. Modern-day cryo-TEMs use Mueller-type sources where the emitter’s shape defines the electron beam shape, typically resulting in a circle. For TEM imaging in a 3-condenser system, the beam current (spot size) is selected by the C1 and C2 lenses, and the source beam width (beam convergence) is selected by changing the strength of the C2 and C3 lenses. The aperture between the C2 and C3 lenses becomes the beam-shaping aperture. When the electron beam cross-over above the C2 aperture is moved (by changing C1/C2 lenses) the beam current changes, whereas when the cross-over below the C2 is moved (by changing the C2/C3 lenses), the size of the beam changes. The post-C2 aperture beam takes on the shape of the aperture’s hole when the beam is spread wider than the aperture. For practical reasons linked to manufacturing and to simpler optical propagation, all apertures have round holes, which in turn create round beams. In this work, we use a square C2 aperture to create a square electron beam profile. We demonstrate its utility on an FFI-capable TEM for near-perfect tiling in montage tomography and increased efficiency in data collection for single particle analysis with minimal loss of resolution.

Using a square C2 aperture, we successfully created a square beam (Figure 1C). First, we adjusted the beam width using the microscope intensity control such that the beam had the same size as the shortest dimension of the sensor. Then, the (post-objective) projection P2 lens was adjusted to rotate the beam square onto the sensor (Figure 1C). Aligning the beam with the sensor ensures that the sensor images the entire sample area exposed to the beam. Since changes in the P2 lens strength changed the image’s rotation, magnification, and defocus; pixel size, image shift, and eucentric focus calibrations had to be redone. The flux on the sensor can be adjusted with spot size and the beam intensity distribution across the illuminated area can be measured to ensure uniform exposures (Supplementary Figure 2). The unique P2 lens state can be stored as a unique magnification entry and added as a separate registry key.

With a square beam, it became possible to tile with minimal overlap to exhaustively image a large field-of-view. This is especially important for *in-situ* tomography, where it is often useful to image large contiguous areas of a specimen, such as a lamella, at high resolution. Acquisition targets can be set along the tilt axis to overlap minimally and therefore reduce any areas on the sample that are exposed to the electron beam in more than one acquisition target. To show this, we tested four data collection schemes utilizing PACEtomo image-shift data acquisition approach (Eisenstein et al., 2023) (Figure 1D,E). The amount of overlap was observed to remain the same at 0° as at high tilts up to 45° (Supplementary Figure 3, rows “Line, no overlap”, “Square, no overlap”, and “Square, overlap”). However, acquisition targets that were set perpendicular to the tilt axis would increasingly overlap at higher sample tilts (Supplementary Figure 3, row “Square, overlap”). To maximize the acquisition area while avoiding the sample overexposure in the direction perpendicular to the tilt axis, a new data acquisition scheme must be developed, where the beam-image shift is proportional to the sample tilt. After data acquisition, a montage for each stage tilt can then be stitched to produce a large field-of-view image, which is then aligned across the tilt series and reconstructed.

In single particle data acquisition, a square beam significantly increases throughput. Using common supports such as UltrAuFoil R1.2/1.3 grids, a square beam could image 85 targets per stage movement (17 holes x 5 targets per hole) versus 34 targets for a round beam (17 holes x 2 targets per hole) (Supplementary Figure 4).

While we observed normal behavior during microscope alignment and coma correction (Supplementary Figure 5), we consistently obtained a slightly worse reconstruction B-factor for the square beam, although with enough particles, the reconstruction still went to Nyquist (Figure 2). With smaller particle sets, we consistently observed a slight loss in reconstruction resolution (~0.1 Å) with the square beam compared to the round beam (Figure 2). The most notable change from the round to the square beam is that the spherical aberration fit increases from 2.9 to 3.1 mm during homogeneous refinement. The increased spherical aberration is most likely linked to the lack of circular symmetry in the phase profile of the beam diffraction from the square aperture. A potential solution is to use larger apertures to lower the diffraction angle and maintain a more uniform phase profile at the sample plane. We also observed that cropping the square aperture micrographs to exclude the unilluminated area of the sensor resulted in higher resolution reconstructions, whereas cropping round aperture micrographs to the same area did not improve the resolution (Supplementary Figure 6). This is likely because the unilluminated area of the sensor negatively influences motion correction and CTF estimation since it does not contain any useful information, and the boundary between the unilluminated and illuminated areas would also be more poorly corrected and estimated.

Non-circular and square beams have been developed for beam-shaping in electron beam lithography, laser micromachining, and medical laser applications. Here we demonstrate the use of a square beam for nearly perfect tiling in cryo-EM and montage cryo-ET. Other non-round beams, such as rectangles and hexagons, can also be considered, which are also optimal profiles for good tiling and imaging efficiency. We note that it is possible to create a rectangular beam by stigmating a square beam; however, this introduces unwanted aberrations into the beam. Furthermore, matching the beam’s and detector’s shapes prevents minor data processing complications associated with having unilluminated sensor areas.

Among optimizations, the mechanical alignment of the aperture orientation is critical to ensure optimal overlap between the sensor and the illuminated area. We envision that the aperture can be mechanically rotated through a redesign of the aperture strip: a worm wheel gear can be installed to physically rotate the square aperture while it is in the liner tube under vacuum during illumination. For this work, we propose a no-cost solution that involves adjusting the P2 lens’ current to induce a rotation of the projected image plane. This introduces changes in the optical system, affecting the image’s magnification, defocus, and rotation, requiring recalibration of the pixel size, eucentric focus, and image-shift matrices.

## Supplementary Material

Supplement 1

## Figures and Tables

**Figure 1.**
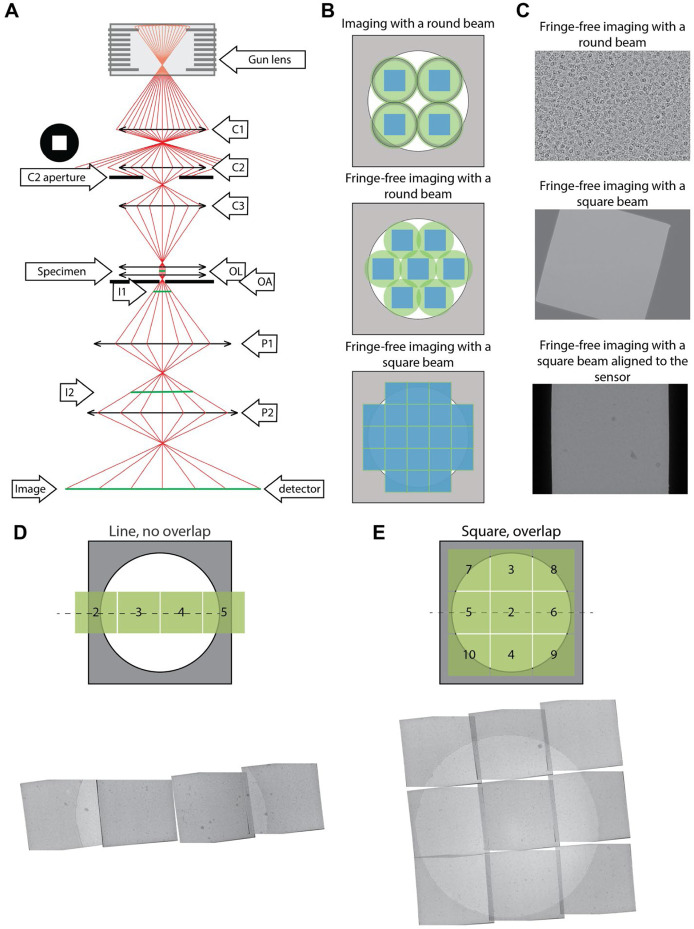
(A) Ray diagram of electrons passing through the column of a TEM. The square aperture is placed at the “C2 aperture” position. C1/2 - condenser lens 1/2, OL - objective lenses, OA - objective aperture, I1/2 - intermediate image plane 1/2, P1/2 - projection lens 1/2. (B) Examples of imaging a specimen (white circle) with different TEM beam setups. The areas on the specimen illuminated by the electron beam are shown in green circles (round beam) or green squares (square beam); the illuminated areas that are captured by the sensor are represented by the blue squares. Fresnel fringes on the circular beam (top image, black rings) in non-fringe-free TEM setups requires the beam to be spread out so the fringes do not fall on the sensor. (C) Example micrographs from an FFI-enabled TEM, taken with a round beam (top), square beam (middle), and a square beam aligned square to the sensor by adjusting the projection lens (bottom). (D) Example PACEtomo data collection schemes with the square beam. We collected data with minimal to no overlap along the tilt axis; or (E) with a 3x3 square montaging setup. Shown are montaged images from the aligned tilt series at 0° tilt. Overlapping areas were identified and aligned by hand and suffer from overexposure to the electron beam.

**Figure 2.**
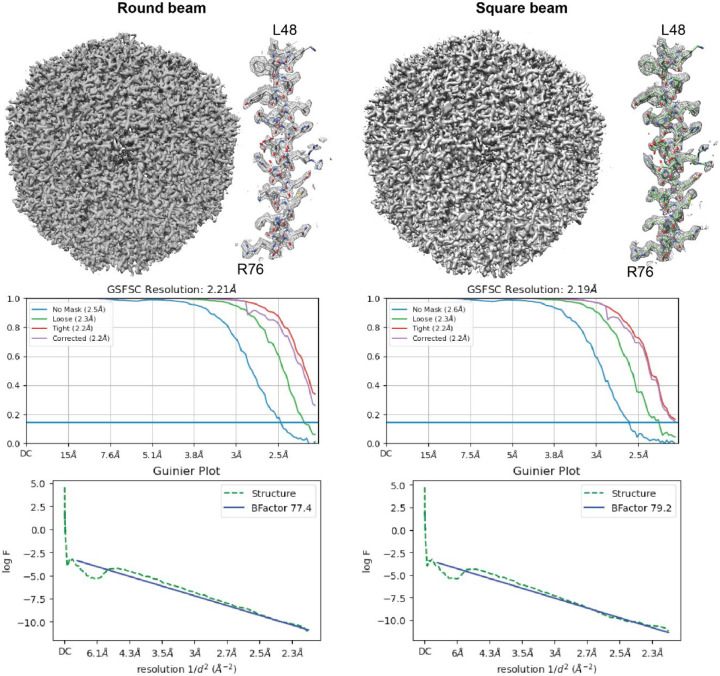
Single particle reconstructions of apoferritin with data collected with a round (left) or a square (right) beam. In both cases, with 120,000 particles, the reconstructions can achieve Nyquist resolution.
