# Supplementary material for: Square beams for optimal tiling in TEM
Source: Res Sq. 2023 Aug 9:rs.3.rs-3220524. Preprint. [Version 1] doi: 10.21203/rs.3.rs-3220524/v1 (PMC10441458; doi:10.21203/rs.3.rs-3220524/v1)
Supplement: Supplement 1 [file NIHPPrs3220524v1-supplement-1.pdf]

Supplementary material

Methods

Apertures with square holes were purchased from Agar Scientific. The apertures were made of platinum, with a diameter of 3.04 mm, a thickness of 0.25 mm, and a square hole of 50 or 100 µm. The aperture was inserted into the C2 aperture strip of the microscope. Prior to insertion into the aperture strip, the square aperture can be plasma cleaned to remove any impurities, then maintained in a sealed container for several days to allow the charge to dissipate for easier insertion into the aperture strip.

For montage tomography, Quantifoil R2/2 300 mesh Cu grids were hydrophilized with a mixture of H_2 and O_2 gas (6.4:27.5 ratio) at 50 W for 30 seconds in a Solarus Model 950 Advanced Plasma System (Gatan). 3 µl of 1 mg/ml "PP7 WT" virus-like particles (VLPs) (Zhao et al., 2019) was pipetted onto each grid, blotted for 3-5 seconds in a Vitrobot at 20°C and 100% relative humidity, then vitrified in liquid ethane. The P2 projection lens was detuned to rotate the square beam square onto the sensor, resulting in changes in the image's magnification, rotation, and defocus. Eucentric focus calibration, pixel size, and image shift calibrations must be performed in SerialEM (Mastronarde, 2005). Tomography data was acquired using PACEtomo scripts incorporated into SerialEM 4.1 beta 13 with a pixel size of 2.12Å/px, exposure dose of 3.4 e^-/ $Å^2$ per tilt, and -45° to 45° tilt range for a total of 31 tilts and total dose of 105 e^-/ $Å^2$ per tilt series. Acquired tilt series were first motion-corrected with Warp (Tegunov & Cramer, 2019) and tilt series were aligned and reconstructed with AreTomo (Zheng et al., 2022) at bin4 and used without additional processing for further analysis.

For single particle analysis, UltrAuFoil R1.2/1.3 300 mesh Au grids were hydrophilized with a mixture of Ar and O_2 gas (26.3:8.7 ratio) at 15 W for 7 seconds in a Solarus Model 950 Advanced Plasma System (Gatan). 3 µl of 8 mg/ml mouse apoferritin was pipetted onto each grid, blotted for 3-5 seconds in a Vitrobot at 20°C and 100% relative humidity, then vitrified in liquid ethane. The P2 projection lens was detuned to rotate the square beam square onto the sensor, resulting in changes in the image's magnification, rotation, and defocus. Eucentric focus needed to be reset on the microscope by adjusting the objective lens, then beam and image shift and scale rotation calibrations needed to be redone in Leginon (Cheng et al., 2021; Suloway et al., 2005) prior to data collection. Pixel size calibration was done in SerialEM on a standard cross-grating replica grid. Energy filter alignments were done per standard protocol, with the entire sensor illuminated. Objective lens astigmatism and coma correction were performed using Sherpa, with the full sensor illuminated. Single particle data were collected using Leginon with either a 100 µm round C2 aperture or a 50 µm square C2 aperture. Data were collected at a pixel size of ~1.08 Å/pixel, a flux of ~30 e/px/s for 2 seconds, equaling a total dose of ~51 e/$Å^2$, with a nominal defocus range of -0.5 µm to -2.0 µm. The square beam was condensed to match the size of the sensor to maximize the data acquisition area, and in the control experiment, a round beam was used with its intensity set to match the flux of the square beam on the sensor. Data were collected using beam-image shift (Cheng et al., 2018).

For single particle data processing, square and round beam data was first motion corrected with patches in cryoSPARC v4.2.1 (Punjani et al., 2017). Full micrographs were then patch CTF estimated, and particles were picked using apoferritin templates. Particles were extracted with a box size of 280 pixels, 2D classified, and 120,000 particles randomly selected for homogeneous refinement. To exclude the unilluminated areas of the sensor because of the condensed square beam, a central square region of the motion-corrected micrographs was cropped out with the IMOD (Mastronarde & Held, 2017) command "trimvol". The same central square region was cropped from both square and round aperture data as a control. Cropped micrographs were then re-imported into cryoSPARC, and the same processing workflow continued as above. Reconstructions from full and cropped micrographs from square and round aperture data were compared.

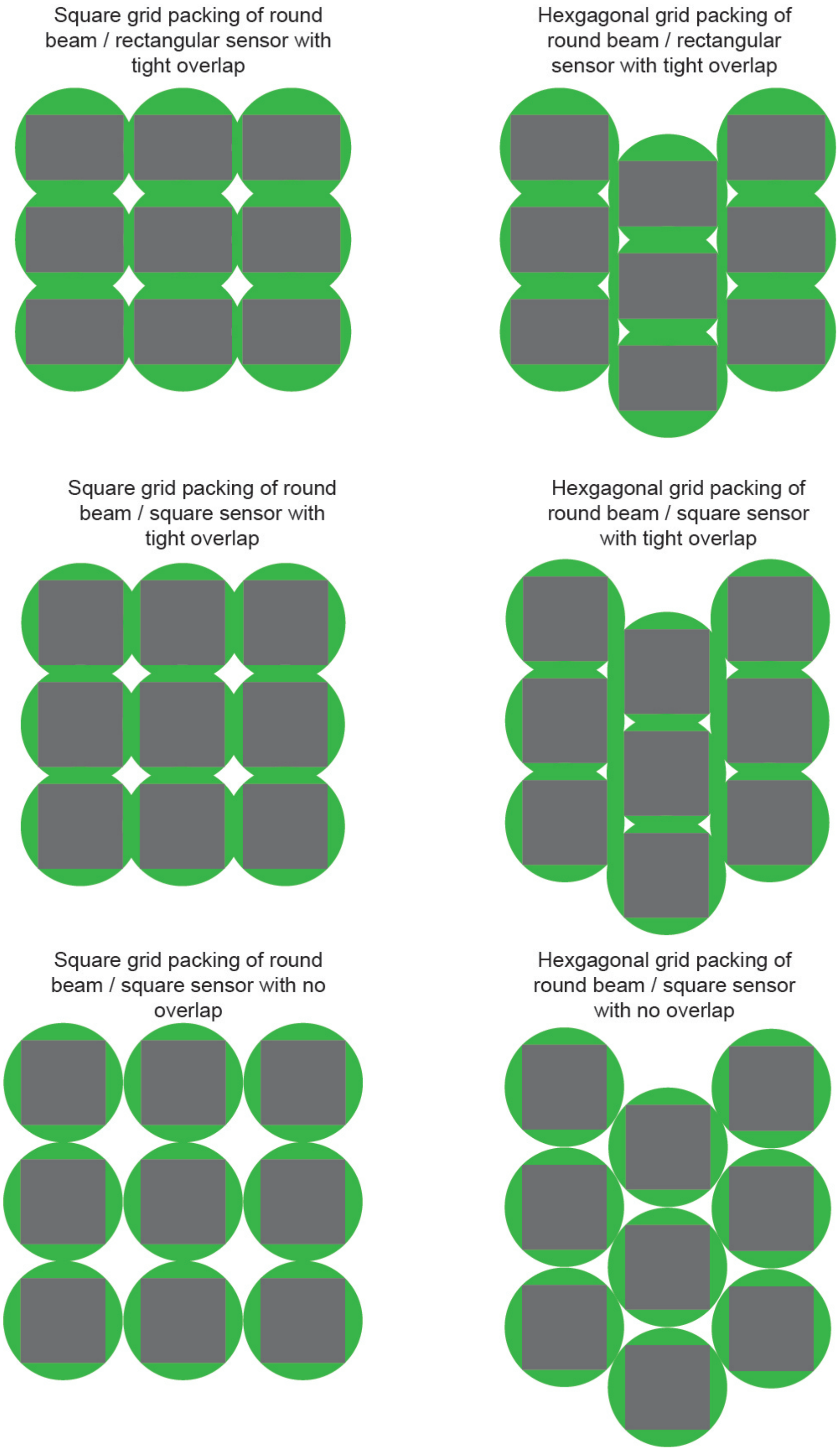

Supplementary Figure 1. Examples of packing circular beams (green) and square sensors (grey) highlighting the gap between the exposed and imaged areas.

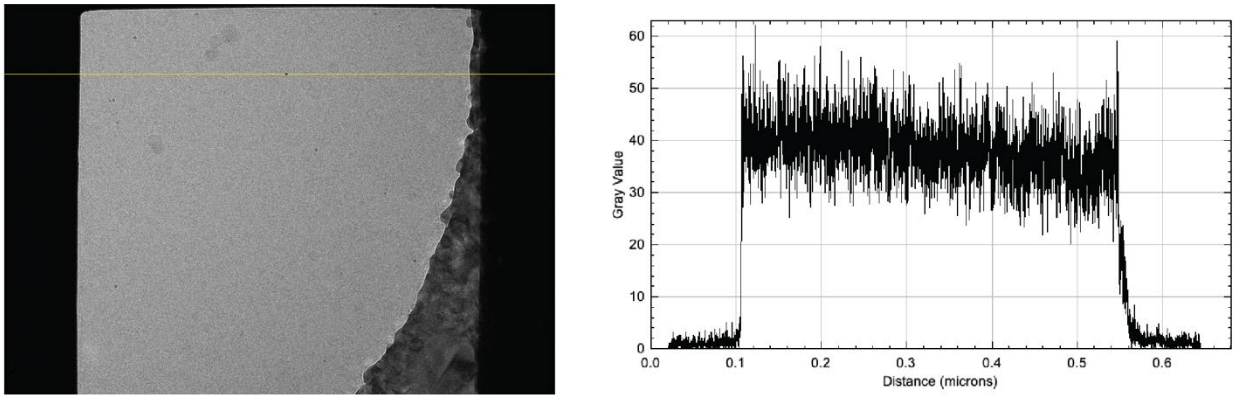

Supplementary Figure 2. Example micrograph taken with a square beam (left), showing a pixel intensity profile (right) for the pixels along the yellow line. Pixel intensity profile was obtained with ImageJ (Schneider et al., 2012).

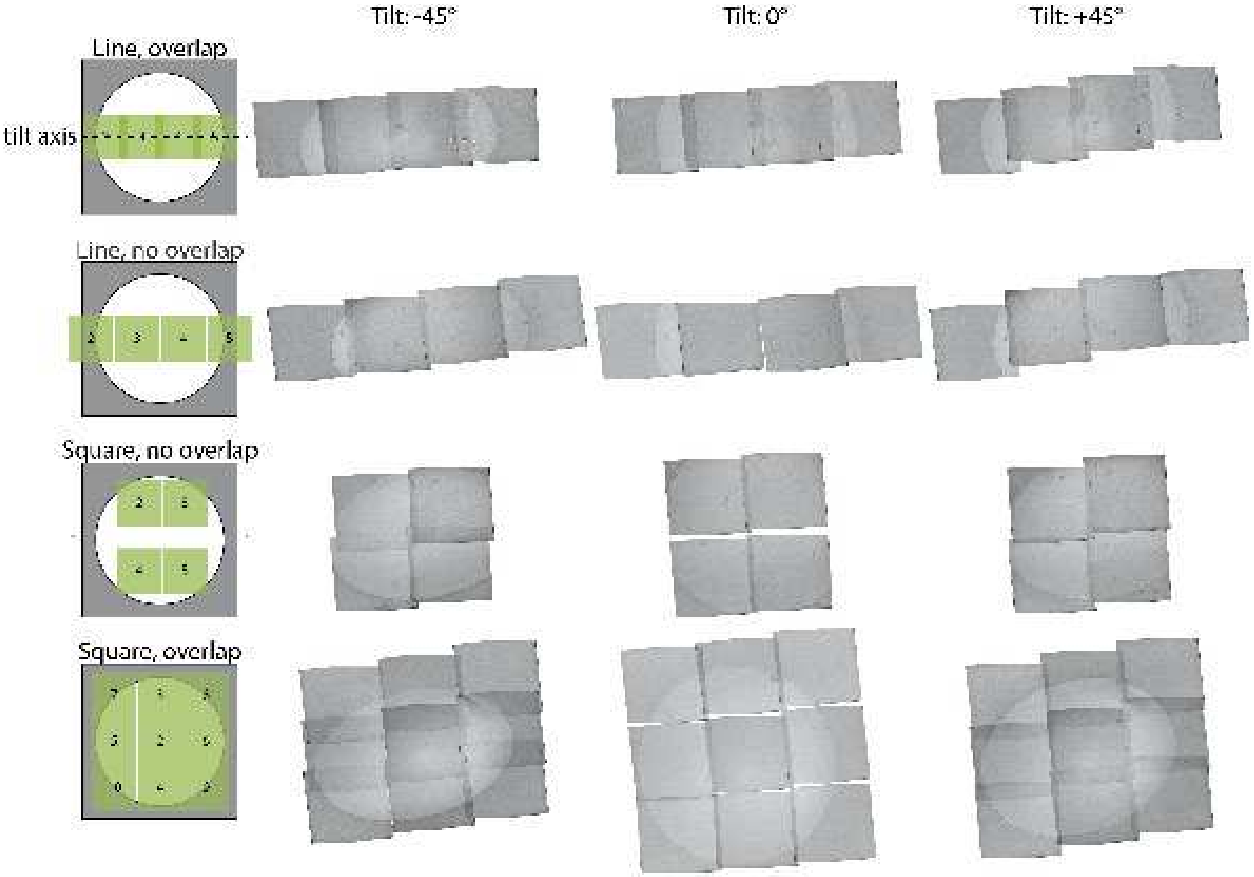

Supplementary Figure 2. Tomography data acquisition schemes with the square beam.

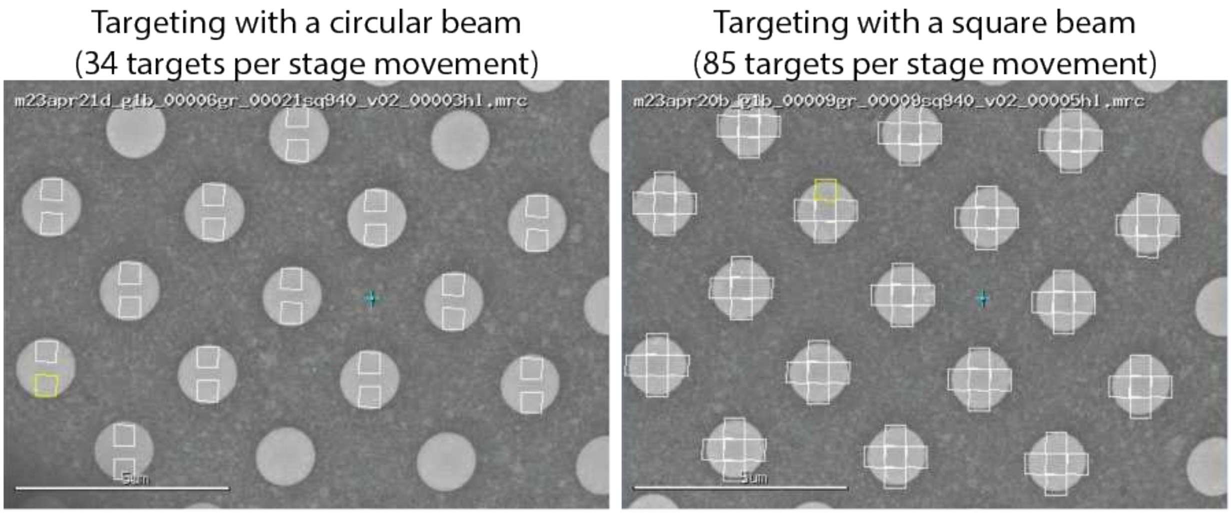

Supplementary Figure 3. When using a round beam and fringe-free imaging, two acquisition targets can be acquired for each 1.2 µm hole (left). In this field of view, up to 34 acquisition images can be taken per stage movement. When using a square beam with perfect tiling, five acquisition targets can be acquired for each 1.2 µm hole (right), increasing the number of images to 85 per stage movement.

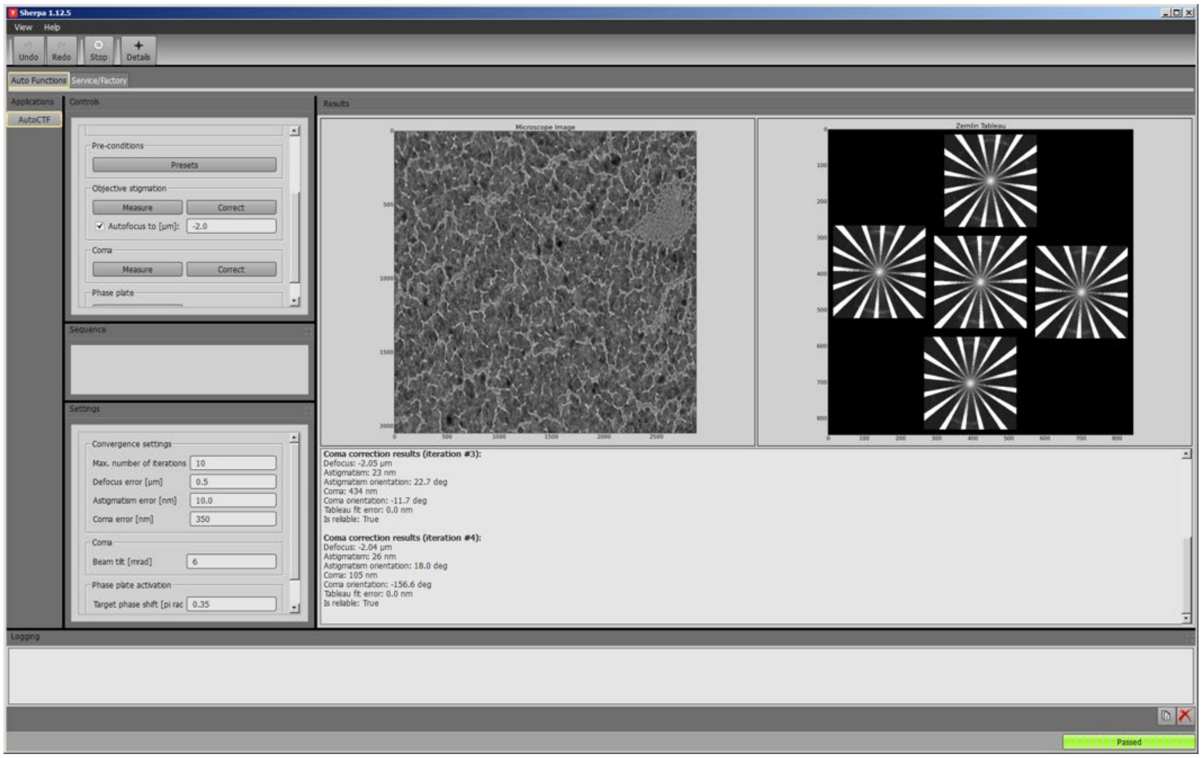

Supplementary Figure 5. Automated coma correction while using a square beam shows it is possible to achieve acceptable levels of coma, directly comparable to using round illumination.

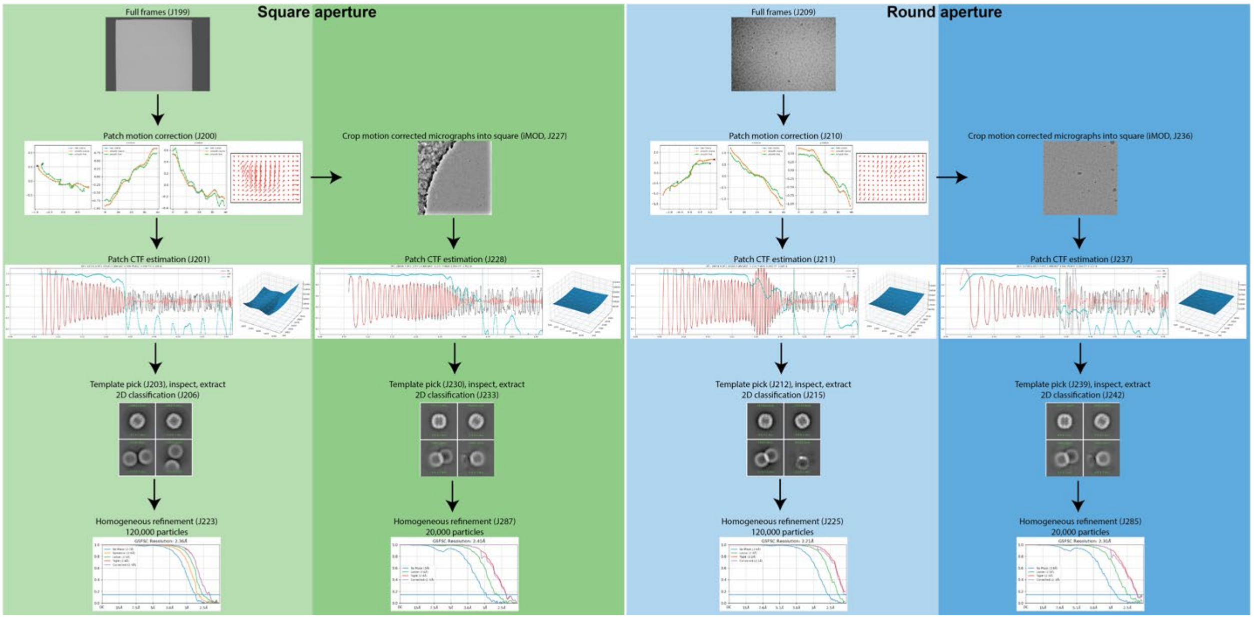

Supplementary Figure 4. Single particle data processing workflow for the square (green) and round (blue) apertures. For each aperture type, the processing workflow uses the full frames on the left, and the processing workflow with cropped micrographs (to exclude the unilluminated areas of the sensor) on the right.
